# An eye tracking based virtual reality system for use inside magnetic resonance imaging systems

**DOI:** 10.1038/s41598-021-95634-y

**Published:** 2021-08-11

**Authors:** Kun Qian, Tomoki Arichi, Anthony Price, Sofia Dall’Orso, Jonathan Eden, Yohan Noh, Kawal Rhode, Etienne Burdet, Mark Neil, A. David Edwards, Joseph V. Hajnal

**Affiliations:** 1 Centre for the Developing Brain, School of Biomedical Engineering and Imaging Sciences, King’s College London, London, SE1 7EH UK; 2 Department of Bioengineering, Imperial College London, London, SW7 2AZ UK; 3 Department of Physics, Imperial College London, London, SW7 2AZ UK; 4 Department of Electrical Engineering, Chalmers University of Technology, 412 96 Gothenburg, Sweden; 5 Department of Mechanical and Aerospace Engineering, Brunel University London, London, UB8 3PN UK

**Keywords:** Biomedical engineering, Magnetic resonance imaging, Translational research

## Abstract

Patients undergoing Magnetic Resonance Imaging (MRI) often experience anxiety and sometimes distress prior to and during scanning. Here a full MRI compatible virtual reality (VR) system is described and tested with the aim of creating a radically different experience. Potential benefits could accrue from the strong sense of immersion that can be created with VR, which could create sense experiences designed to avoid the perception of being enclosed and could also provide new modes of diversion and interaction that could make even lengthy MRI examinations much less challenging. Most current VR systems rely on head mounted displays combined with head motion tracking to achieve and maintain a visceral sense of a tangible virtual world, but this technology and approach encourages physical motion, which would be unacceptable and could be physically incompatible for MRI. The proposed VR system uses gaze tracking to control and interact with a virtual world. MRI compatible cameras are used to allow real time eye tracking and robust gaze tracking is achieved through an adaptive calibration strategy in which each successive VR interaction initiated by the subject updates the gaze estimation model. A dedicated VR framework has been developed including a rich virtual world and gaze-controlled game content. To aid in achieving immersive experiences physical sensations, including noise, vibration and proprioception associated with patient table movements, have been made congruent with the presented virtual scene. A live video link allows subject-carer interaction, projecting a supportive presence into the virtual world.

## Introduction

Magnetic Resonance Imaging (MRI) can safely provide non-invasive yet detailed images of the human body and thus is now an integral tool in both clinical practice and research. However, the MRI scanner environment is noisy and claustrophobic, making it challenging to tolerate for many people^1^, particularly those in vulnerable populations such as children or those with cognitive difficulties. Anxiety is further compounded by the fact that the patient or subject must lie by themselves inside the scanner bore, and usually cannot see or hear a carer or companion to receive reassurance during the examination. As a result, scanning failure rates are as high as 50% and 35% for children between 2–5 and 6–7 years respectively^2^. When clinically indicated, sedation or anesthesia are traditional ways to alleviate these problems, but the potential physiological impacts of these interventions cannot be neglected. Commonly used methods to reduce anxiety prior to the examination include providing written information^3^, video demonstrations^4^, telephone conversation^5^ and even mock MRI^6^. However, these do not change the examination process itself. In bore audio-visual systems are becoming more common as options provided by the scanner manufacturers or add-on devices, and do provide distraction, but the subject being examined remains acutely aware of their surroundings and the stresses associated with these.

The immersive capabilities of Virtual Reality (VR) technology have been successfully exploited in clinical applications such as those related to anxiety disorders and phobia therapy^7,8^. It is also being deployed as a tool for desensitization and habituation in the preparation phase prior to MRI examination. Examples include a mobile based VR app, applied to help educate patients about MRI and simulate the experience of actually being scanned^9^, and a more complex PC based VR system for patient preparation, which allows users to navigate in a virtual scanning room and interact with equipment^10^. The latter study shows 86% of participants found VR simulation to be a feasible and accessible alternative to mock scanning to prepare patients for MRI.

Studies using VR to improve the experience of the MRI examination itself so far remain limited, perhaps because of the significant challenges inherent to achieving an MRI compatible VR system. To achieve a visceral feeling of being in a virtual world, it is common to achieve dynamic control of the user’s viewpoint using head mounted displays (HMD) and motion tracking combined with free, and often exaggerated, head movement. Head motion is generally undesirable in MRI, particularly when imaging the brain where it can significantly impair image quality and interpretation. Moreover, unmodified HMDs may not function in a high magnetic field environment, may pose safety risks, may be incompatible with MRI receiver coils and are likely to cause image artefacts. For children, there can be additional difficulties in orienting and navigating using HMD because children are more sensitive to restrictions in the field-of-view than adults^11^. Other key components of standard VR systems, such as controller wands, motion trackers and haptic displays, may also be contra-indicated or at the least require adaptation for use in the MRI scanner environment. In addition, the typical subject pose during imaging, supine and remaining as still as possible whilst constrained inside the relatively narrow scanner bore, is not compatible with usual VR software interfaces and their means of producing interactive and immersive experiences for the subject. The MRI examination is also typically accompanied by noise and vibration, which can both add to stress levels and detract from the sense of immersion into a virtual world.

Audiovisual systems are commonly used during MRI examinations both as a method of distraction and as a means with which to present stimuli for characterizing the associated patterns of brain activity using functional MRI (fMRI). Whilst these typically consist of headphones and mirrors mounted on the head coil (which reflect a video projected from outside the examination room or on a separate display), there are emerging MR specific entertainment systems that focus on improving patient experience, including some which offer stereoscopic 3D displays. These systems can offer user choice and even a limited form of VR through presenting diverting scenes (flower meadows, sky or seascapes etc), films or documents to be read during the MRI examination. The role of immersion, presence, and interactivity in VR is analyzed in^12^, which shows interactivity is the key feature that influences presence and immersion. Although a stereoscopic display is commonly deployed in full VR systems, this feature can cause visual discomfort and fatigue and makes interaction challenging due to associated difficulties with depth perception^13^.

Over the years, eye tracking techniques have contributed to many disciplines in clinical neurology and neuroscience^14,15^. There are already many MRI compatible, in-bore, eye tracking solutions which provide pupil and gaze data, but these aim for analysis of visual attention and behavior rather than enabling interaction. Recently, eye tracking has also become a desirable feature of advanced VR systems to leverage the benefits of both technologies and achieve more immersive and user-friendly experiences^16,17^. In VR games, eye tracking is mainly used as an accommodation for the existing game by replacing, reducing or cooperating with mouse-based operations (such as gaze based aiming, camera view control etc)^18^. For MRI application specifically, shifting from tracking head motion to eye tracking to control the visual scene and enable interaction could have advantages as it not only removes the need for an additional external controller but should also reduce subject head movement.

We hypothesize that completely replacing the visual scene seen by the subject (patient or research volunteer) with tailored VR content that is immersive and interactive could achieve a radically different subjective experience, which could mitigate claustrophobia and allow vulnerable groups, such as children, to be examined more effectively. Exclusion of peripheral visual cues that anchor the subject to their physical situation would allow the VR experience to more completely replace the sensation of being inside an MRI scanner. Further, by giving the subject control and providing fluid communication with a carer outside the scanner, such a system could potentially transform the MR examination from a passive, boring and isolating experience into an active, engaging and interactive one. In addition, making the visual and auditory environment perceived by the subject congruent with incoming physical stimuli and proprioception could create a holistic integrated sense experience. In this paper, we describe a VR system designed to deliver on these aspirations. Such a system should allow the subject to control their VR environment without inducing excessive subject motion. We present a prototype system with user interaction accuracy and robustness. Central to this is a gaze based user interface (UI), which must be responsive and reliable, remaining so despite any incidental changes in head pose during the MRI session.

## System overview

**Figure 1:**
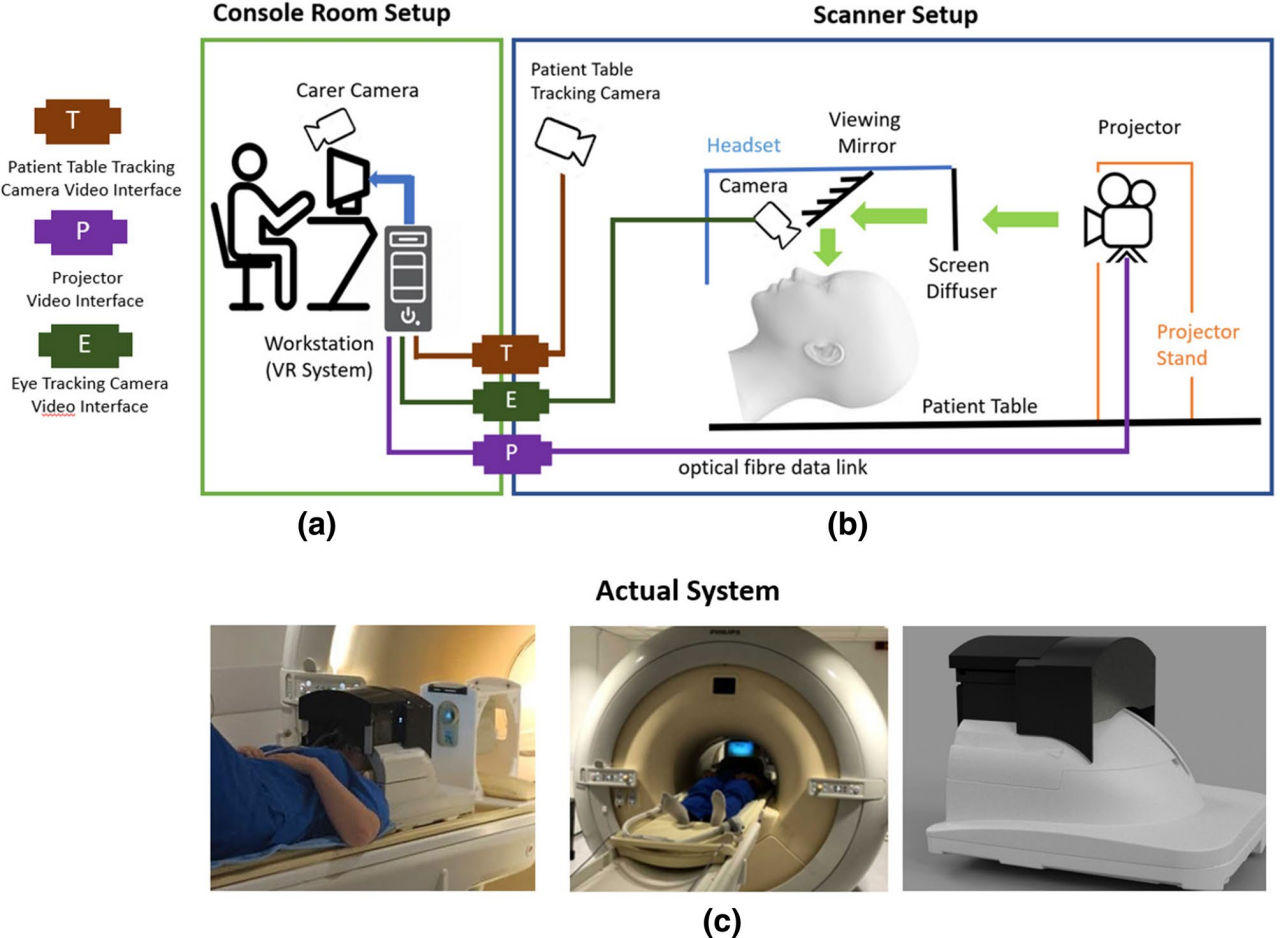
System schematic overview. Illustration of hardware system setup in the scanner and console rooms. (**a**) The console room setup includes the workstation running the VR system, eye and table tracking modules and carer communication system. (**b**) The in-scanner hardware mainly consists of a MR compatible projector, custom designed VR headset and patient table tracking camera. (**c**) Views of the system in scanner and headset design.

To provide subjects with an immersive VR environment, we developed a coil mounted VR headset (Fig. 1) which mates precisely with a standard MRI head coil (in the prototype this is a 32-channel head coil for a Philips Achieva 3T system). The headset is designed to be light tight so the subject cannot see their surrounding environment at all and is therefore unaware/cannot be distracted by visual reminders of their position inside the MRI scanner bore. To avoid either static or radiofrequency (RF) magnetic field disturbances all components remain outside the subject space within the head coil, and with the exception of two MRI compatible cameras (see below), the coil mounted assembly was made completely of plastic (Acrylic and Polylactic Acid) using 3D printing. The design ensures that the subject can be positioned in the head coil without any additional alignment steps and without placing any components in close proximity to the subject’s face.

Content for the VR system is projected onto a diffuser screen by a MRI compatible projector with optical fibre data link (SV-8000 MR-Mini, Avotec Inc, Florida), which is placed beyond the head coil on the patient table (Fig. 1b). The diffuser screen is viewed in transmission via a silvered acrylic reflector. Two MR compatible video cameras, one for each eye, are located within the VR system just inferior to the acrylic screen. The cameras (12M-I, MRC Systems, Heidelberg) each have an infrared illuminator diode and are fitted with 6 mm lens to provide a sharp image of the eye region (Figs. 3, 7). In addition a standard wall mounted camera in the MRI examination room is used to monitor the patient table position to provide data needed for a synchronized motion experience in the VR environment during any patient table movements.

At the start of the examination the subject is positioned in the head coil as for a normal brain MRI scan. Standard ear plugs and a pair of optical, noise cancelling headphones complete with microphone (OptoActive II, Optoacoustics Ltd, Israel) are put in place. The VR system is then positioned on top of the head coil, locking into place by precisely mating with the eye-cutouts in the coil. The projection system is immediately live, providing immersive content and from that point onwards, the VR experience is continuous until subject removal at the end of the examination. The system is designed so that if desired, the subject can be positioned and the VR system made operational while the patient table is away from the scanner, so that it is feasible for the subject never to see the MR system at all. The projector, headphones and microphone are all fed by long (> 20 m) optic fiber links to facilitate this. Purpose designed VR content takes the MRI scanning procedure and subject’s physical status (face up supine view) into account.

A communication system is provided so that the subject can interact at will with a companion or carer at any time during their examination via a webcam with microphone and a display monitor installed in the console area. The system allows two-way audio communication and the subject can summon at real-time video of the companion/carer when desired, which is streamed directly into the VR system view. The companion/carer can provide assistant control using a handheld mouse and is presented with the same screen as the subject, plus a continuous video display showing the subject’s eyes.

A core requirement for the VR system is an interaction interface that gives the subject control while not encouraging them to move. The proposed system uses gaze tracking for this purpose, providing a means with which to navigate through the virtual world, to select content (such as films, games etc), to play games and to initiate/terminate a video link to their companion/carer.

**Figure 2:**
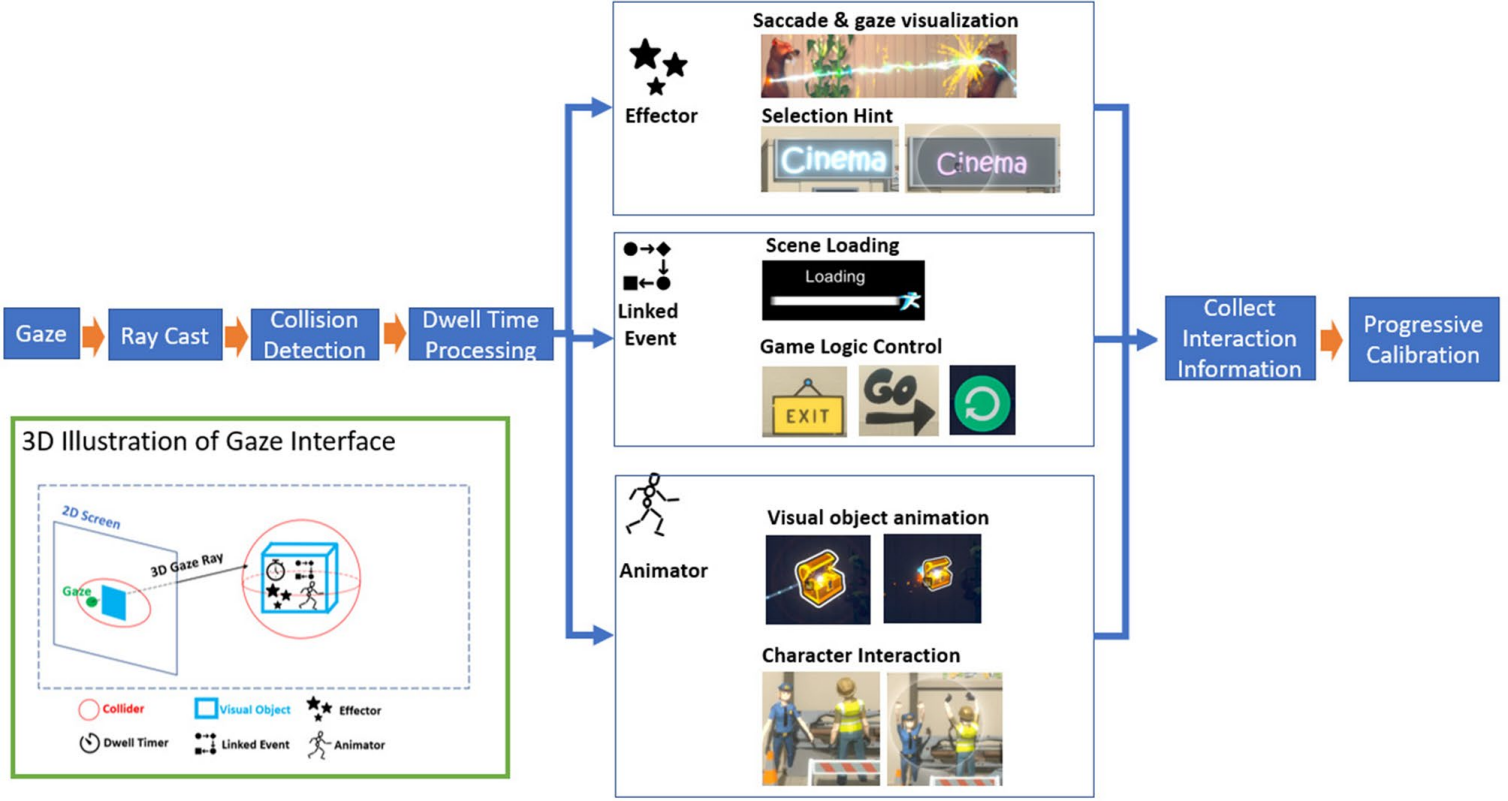
The mechanism of the gaze-based interface and illustration of interactive objects in VR system. The interface converts the estimated gaze coordinates on the display screen into a 3D ray to perform target collision detection. Interaction in the virtual world is based on dwell time and target components.

A central challenge for gaze based interfaces is the so called Midas touch problem^19^, in which fixation on an interface element results in immediate activation even when the user has no such intention. With this in mind, the main design principle of our gaze interaction interface is based on the theory of the brain having two visual processing mechanisms: a ventral stream (“vision-for-perception”) and a dorsal stream (“vision-for-action”)^19^. These two streams are responsible for the quick perception of an object’s location (ambient fixation) and object identification (focal fixation) respectively. To follow this dual perception pattern, the interactive targets in our scene normally have a landing zone larger than the associated visual shape to capture ambient fixation. As soon as the gaze alights on the landing zone for a target, a prompt is provided that indicates the option to initiate an action and feedback on progression towards the start of the action is provided. As the dwell time increases, the visual shape of the target will shrink which is used to transform from ambient fixation to focal fixation. In the beginning of our system, each selection target has a landing zone that is initially large but gradually reduces over time as the gaze prediction model becomes more robust and accurate. Interactive objects consist of six basic components: visual object, collider, dwell time counter, effector, animator and linked event (Fig. 2). The gaze location is transferred from the 2D display geometry to the object and its collider using a 3D ray cast from screen gaze point into the 3D virtual space beyond. The visual object is the actual item displayed in the VR environment. The effector and animator generate visual effects (such as highlight, fade, dissolve, particle effect etc.) and animations (such as motion captured animation, shrinkage etc.) of the visual object when the subject’s gaze dwells on the object’s collider (landing zone). The linked event is triggered when the gaze dwell time exceeds a given threshold. The linked event can be a UI function (button trigger, menu switch, scene loading etc.) or game events (game objects spawn and hide, player teleport, character behavior trigger etc.). Therefore together, the combination of a required dwell time, visual feedback and increasing gaze prediction accuracy all help prevent the Midas touch problem from occurring in our system.

To provide secure control of the system and create an immersive experience, the gaze estimation algorithm needs to be accurate and robust against subject head movement, which will occur to some degree in any examination. Additionally, it should feel natural and not result in strain during prolonged usage. This is fundamental, as maintaining a continuous sense of control is vital to maintain immersion in the proposed system as it represents the bridge between the user and the VR world. To achieve these requirements, an adaptive approach to gaze tracking that features progressive calibration refinement throughout the examination has been developed.

**Figure 3:**
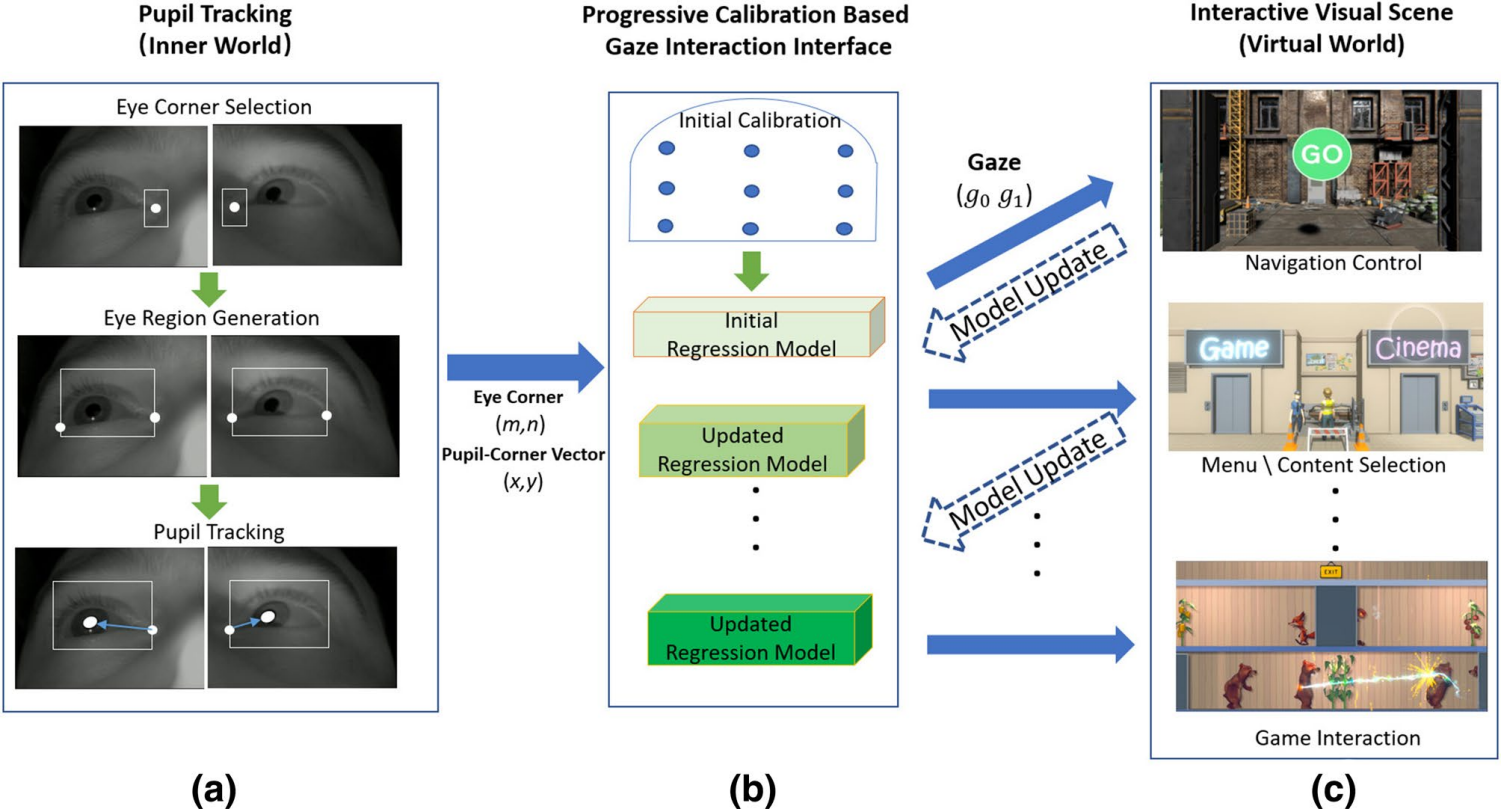
Illustration of the system pipeline: the eye tracking stage tracks the eye corner and pupil location for each eye. The gaze estimation module uses the eye tracking results to build an initial regression model which is then updated by interactive calibration. The gaze information is fed into the VR system to trigger interactions. Interactive progressive calibration is executed in the background when interactions are triggered. This mechanism can progressively improve the gaze regression model accuracy without changing the user experience.

## Gaze tracking engine

In general, eye tracking systems can be categorized into head-mounted (wearable) and remote devices^20^. The majority of these systems are based on head-mounted designs which make eye tracking less challenging as there is no influence of head movement. Our scenario is entirely distinct as it requires the combination of VR with gaze estimation from a remote eye tracking system in a highly circumscribed environment.

Remote eye tracking systems usually require the detection of facial features to localize eye regions and to assess head pose to help determine gaze direction. In our scenario, as the subject is lying in a highly constrained environment in which there are only minor changes in lighting conditions, we are able to use both head position and appearance of the eyes across the duration of use. However, a potential drawback of making our system entirely unobtrusive with a remote (non-invasive) design is that full facial context is not available due to the shape of the receiver head coil and limited space inside the MRI scanner bore (as can be seen in Fig. 3). Previously described realtime head motion tracking methods based on limited facial features are not suitable and so most MRI compatible eye tracking systems require a subject to stay relatively still during eye tracking^21^. One possible solution is to use a markerless optical head tracking system using facial features^22^. However, this system requires a complex stereoscopic setup (implemented with 4 cameras) and cannot be used in realtime, making it unsuitable for our application.

Conventional gaze prediction methods can be classified into: 2D regression/feature^23–26^, 3D model^27–29^, cross ratio^30–32^ and appearance based methods^20,33–35^. The 2D regression/feature-based methods directly map informative local features of the eye to 2D gaze coordinates. In addition to full facial context, the majority of these methods use a pupil center corneal reflection (PCCR) vector as an input to estimate gaze, which relies on the fact that a light source reflected from the cornea will remain relatively stationary when the eye moves. However, a significant drawback of corneal reflection based methods such as these is that they require good subject cooperation to ensure that the reflection appears within the subject’s corneal region. This is also more challenging for near eye light sources as head/eye motion could make the corneal reflection disappear. Although complex light source setups can potentially improve this, they also increase the risk of causing an undesired red eye effect (a bright disc against the surrounding iris due to light reflecting directly from the retina)^36^ when subjects look at a specific angle, which significantly influences pupil tracking accuracy and robustness. This is a particular concern in our scenario, as there is illumination from both the screen and viewing mirror in addition to the LEDs, all of which are within 200 mm of the face, resulting in more intense local specular reflections on the subject’s eyes. Whether these reflections overlap the cornea is unpredictable as it is dependent on head pose, eye appearance and screen brightness. Therefore, this uncertainty about corneal reflection makes the 2D regression and feature-based models, and the cross-ratio based methods all unsuitable for our purposes. Furthermore, a further limitation of 2D regression/ feature-based methods is that the normal calibration routine cannot provide enough training data to generate a robust regression model to accommodate all possible behaviours, which means the initial fixed regression model will likely only work well in a static pose. Therefore in our case, a fixed regression model would not be suitable as it would not be able to maintain accuracy when slight head motion inevitably occurs. Alternative appearance-based methods require large amounts of data to achieve robust performance, but even then do not provide advantages in accuracy^20,33^. We therefore chose to adopt a 2D regression-based method, but describe a novel adaptive calibration model to prevent the initial calibration from becoming invalid should the subject position change during use. A detailed comparison between state-of-the-art gaze prediction methods and our method can be found in section “Comparison with state-of-the-art research”.

### Head motion estimation and pupil detection

Estimation of head motion in real time can be achieved by tracking markers attached to the subject’s head^37^. However, this is not desirable in the clinical context and when imaging vulnerable subjects as applying makers is intrusive, adds difficulties to subject preparation and can result in hygiene problems. Markerless head motion estimation typically relies on detecting specific facial features such as the eye corners, nose tip and/or mouth corners^38^. Of these, only the eye corner is available in the present system, however, traditional feature-based detection methods^39^ do not provide stable and reliable results when used in isolation. To achieve stable tracking we adopt a state-of-the-art tracking method based on a discriminative correlation filter with channel and spatial reliability (DCF-CSR)^40^. DCF-CSR uses spatial reliability maps to adaptively adjust the filter support region from frame to frame, and deploys online training to handle target object appearance changes. The tracking accuracy and stability of this approach has been shown to be very competitive compared to other popular tracking algorithms such as MOSSE^41^, GOTURN^42^, KCF^43^, BOOSTING^44^, and MEDIANFLOW trackers^45,46^.

In the present implementation, the inner and outer eye corners are manually marked using the live video streams when the subject is ready to start (Fig. 3a). A small bounding box centered on the inner eye corner mark and an eye region bounding box with a fixed width: height ratio based on the positions of the inner and outer corners are then automatically generated. The positions of both boxes are updated from frame to frame using the DCF-CSR algorithm. Offsets of the eye corner box provide information used to record changes in head pose, while the whole eye region bounding box specifies where pupil detection and segmentation is to be performed. Importantly, it is not necessary for the manual marking to be accurate as the eye corner is a region rather than a pixel-level feature which allows it to be both selected quickly and negates the requirement for highly accurate placement.

Given high resolution input images (640x480 pixels for the eye region alone) and a stable lighting environment (consistent relationships between illuminators and the subject’s face), pupil segmentation is a simple task that can be reliably achieved using adaptive intensity thresholding, morphology (dilation and erosion), edge detection and ellipse fitting^47–49^. The system extracts coordinates for the center of the pupil, ($px^t$, $py^t$), and inner eye corner, ($m^t$,$n^t$), from each video frame labeled by time, *t*. From these, relative pupil coordinates are defined as $x^t = px^t - m^t$ and $y^t = py^t - n^t$, allowing a 4D feature vector, $\mathbf{e} ^t = (x^t,y^t,m^t,n^t)$, to be assembled for each eye.

### Adaptive gaze prediction

Gaze prediction requires a model that maps the 4D feature vector $\mathbf{e} ^t$ to the current 2D gaze point coordinates, $\mathbf{g} ^t$, on the display screen. It is common to use polynomial regression for this purpose, in which case the model can be expressed as:

1 $$\begin{aligned} {\mathbf{G}} = ({\mathbf{g}} ^1, {\mathbf{g}} ^2,... {\mathbf{g}} ^M)^T = {\mathbf{Vc}} \end{aligned}$$

where $\mathbf{g} ^t \in \mathbf{R} ^{2\times 1}$ and $\mathbf{G} \in \mathbf{R} ^{M\times 2}$. $\mathbf{G}$ is the target matrix of coordinates from *M* gaze fixation events, $\mathbf{c} \in \mathbf{R} ^{N\times 2}$ is a matrix of model coefficients (2*N* unknowns need to be solved) and $\mathbf{V} \in \mathbf{R} ^{M\times N}$ is an observation matrix whose rows are constructed from $\mathbf{e} ^t$. For $M > N$ the model can be solved as:

2 $$\begin{aligned} \mathbf{c} = (\mathbf{V} ^T\mathbf{V} )^{-1}{} \mathbf{V} ^T\mathbf{G} \end{aligned}$$

Finding the best model, including accommodating different head positions, is quite challenging. Prediction accuracy has been studied^50,51^ in over 400,000 models using polynomial expressions up to fourth order, and no single equation was significantly better than the rest for general usage. In our case, head movement is expected to be small due to the constraints imposed by the head coil, with predominately gradual pose changes as the examination proceeds, plus perhaps sporadic jerky movements. Quadratic polynomial models have been found to achieve good accuracy when the head is relatively static^20^. Thus we have explored quadratic and cubic models for the pupil-corner vector $(x^t,y^t)$, with added terms in $m^t$ and $n^t$ to account for head motion. As will be seen later, a quadratic model with linear eye corner terms was found to be the best, resulting in the following expression:

3 $$\begin{aligned} \begin{pmatrix} \mathbf{g} ^1 \\ \mathbf{g} ^2 \\ ... \\ \mathbf{g} ^t \end{pmatrix} = \begin{pmatrix} x^1 &{} y^1 &{} x^1y^1 &{} (x^1)^2 &{} (y^1)^2 &{} m^1 &{} n^1 &{} 1\\ x^2 &{} y^2 &{} x^2y^2 &{} (x^2)^2 &{} (y^2)^2 &{} m^2 &{} n^2 &{} 1\\ ... &{} ... &{} ...&{} ...&{} ...&{} ...&{} ...&{} ...\\ x^t &{} y^t &{} x^ty^t &{} (x^t)^2 &{} (y^t)^2 &{} m^t &{} n^t &{} 1 \\ \end{pmatrix} \begin{pmatrix} \mathbf{c} _1 \\ \mathbf{c} _2 \\ ... \\ \mathbf{c} _8 \end{pmatrix} \end{aligned}$$

The final gaze location is taken to be the mean of the locations predicted by the separate models for each eye. An option to differentially weight the two models was tested, but was not found to be helpful.

The conventional approach would be to solve for the model coefficients in equation (3) using a calibration procedure. This presents two challenges in practice: 1) The calibration task, usually fixation on a sequence of targets on the display screen, can be challenging, stressful and may be tedious to complete; 2) Although it is trivial to span the two degrees of freedom for the display screen, it is not so simple to systematically gather data on the effect of changes in head pose, especially as for application with MRI, it is desirable for the subject to remain as still as possible. As already noted, we adopt an adaptive approach which is achieved by taking advantage of a core feature of the proposed user interface, in which the subject makes selections by holding their gaze on fixed targets. The aim is to keep the subject comfortable and focused on their own self-selected activities. Each time a selection is made, a new piece of calibration data becomes available, and is used to add rows to the matrices $\mathbf{G}$ and $\mathbf{V}$. To achieve robustness, each selection target has a landing zone (the region of the VR space associated with it) that is initially large, but gradually reduces over time as the gaze prediction model becomes more robust and accurate. Incidental head movement may temporarily reduce model accuracy, and if this occurs, the landing zone size is temporarily increased again.

To initialize the model, a minimal set of fixations are required to estimate its parameters (8 for the quadratic model including head motion). We follow the widely used standard 9 calibration point procedure^52^, in which a sequence of points appear one at a time on an otherwise dark screen (Fig. 3b). The size of the calibration points gradually decreases during the gaze fixation period (see supplementary video S1); this dynamic element is designed to encourage the subject to remain fixated on the target and echoes a feature of the main UI. During this period, frame by frame data for the eye features are accumulated into a buffer. This is done because even when a subject is looking at a static object with the head relatively still, there are still detectable pupil movements as a result of tremor, drifts, and microsaccades. The median value of the buffered data is used for the feature vector, $\mathbf{e} ^t$, corresponding to the target gaze coordinate $\mathbf{g} ^t$. The duration of fixation and the order in which the targets were presented (sequential or random) were explored to optimize performance and acceptability. After the initial minimal set of fixation points, further targets are presented as part of an introductory scene that is used to acclimatise the subject to interacting with their virtual environment, to teach them how the UI works and to refine the gaze estimation model (see supplementary video S1). During this scene a sequence of targets is presented at diverse locations, so as to span the available display region. These targets are made highly prominent to command attention, they change when gazed at, and they trigger very obvious events to establish the modus operandi.

## System implementation

The system is a fusion of real-time computer graphics and computer vision technologies, composed of three independent modules: the VR simulation (including the carer interface as a video input), eye and patient table tracking systems (see supplementary video S1). A server-client model is adopted in which the VR system receives and processes data from the eye tracking and patient table tracking systems. This type of design enables the full system to be easily extended by adding more types of clients for different tasks without influencing other modules. The VR system and carer system are developed using the Unity game engine development platform (Unity 2019.4); the eye and patient table trackers are based on Python; and OpenCV is used as the main computer vision library. The system logs all video streams and records all extracted features with time stamps to provide system diagnosis and for use in studies of the subject, for example using fMRI.

### User Interaction (UI) design

#### Menu and general interaction design

UI interaction is based on selection by gaze fixation. To promote deliberate actions, avoid false selections and provide feedback to the user, targets which trigger actions receive a highlight accompanied by a sound effect when the subject’s gaze point resides within a defined landing zone. If the gaze remains in the landing zone, the visible menu item will shrink until, after a preset dwell time, selection is confirmed by the menu item vanishing and the resulting action commencing. The dwell time for a selection action and landing zone size are configured by an external parameter file to allow tuning and optimization. Note that the size of the landing zone is selected to achieve a balance between robustness to errors in the current gaze estimation model and specificity of gaze selection. Landing zones are used as a filter and do not play any role in model estimation (see section “Adaptive gaze prediction”).

At the heart of the UI is a lobby scene from which the subject can select activities using a menu system with two layers: the first layer is the content category (Games, Cinema) and the second layer (sub-layer) can provide detailed content for each category. In the lobby, menus have five types of buttons for navigation and selection: entry, exit, next, previous and select. In addition to UI selections, the system also supports character and object interaction as well as enabling an extended view and maintaining a clean UI. Character interaction triggers responses from virtual figures based on brief glances and character teleport (navigation) can be triggered by fixation. Environment/object interaction uses the same cues to trigger diverse events such as particle effects and object highlighting. An extended view feature provides a scene panning effect on the field of view based on gaze point position on the screen instead of having the camera view completely locked to the forward move direction. To maintain a clean UI and avoid distracting clutter some items can be dimmed or made transparent until the subject’s gaze is directed to their location, when they re-appear ready for interaction or selection.

#### Maintaining congruence with physical stimuli and proprioception

**Figure 4:**
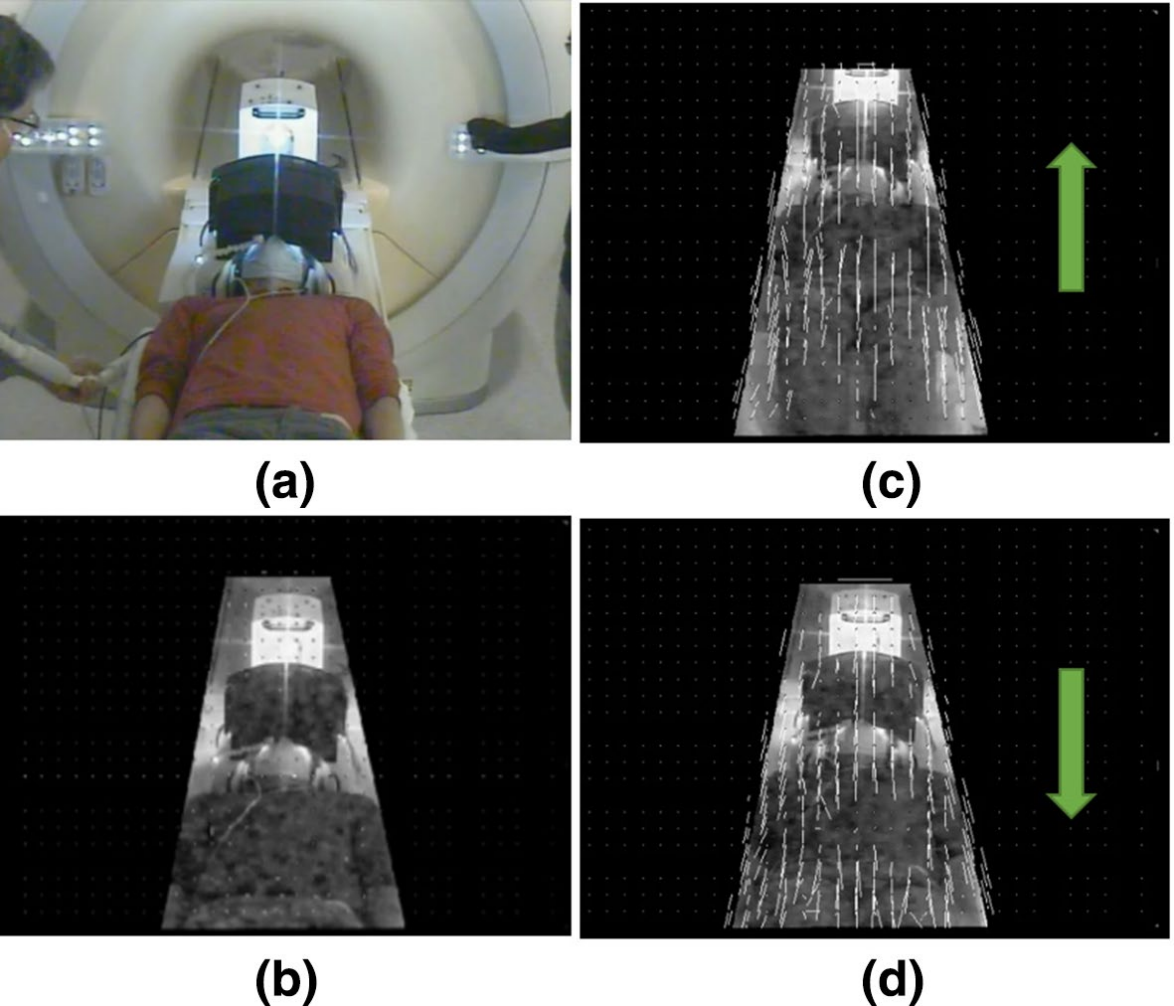
Illustration of patient table tracking: (**a**) the original frame and (**b**) masked grayscale frame. (**c**,**d**) table motion vectors derived from optical flow at sampled pixels in the masked region when patient table moves.

In addition to audio-visual stimuli provided by the VR system, the subject experiences physical sensations in the form of noise and vibration during MRI scanner operation and sensations of motion during patient table movements. To prevent these incongruent physical stimuli from impairing the sense of immersion, these aspects of perception are integrated into the VR environment with elements of the visual scene designed to provide explanatory causes for physical sensations. For example, the subject sees characters who are repairing the virtual scene using tools likely to cause noise and vibration. When the subject gazes at these characters, they apologize for the disruption they are causing. Patient table motion is also tracked using the video stream from the patient table tracking camera (Fig. 1b). Video frames are first cropped to the region of the patient bed using a predefined binary mask (Fig. 4) and then Gunner Farneback’s algorithm^53^ is used to compute the optical flow for all remaining pixels. Subject motion is estimated by averaging all the optical flow vectors to provide a 2D motion direction and speed, which is fed to the VR system to produce motion synchronization of the visual scene.

### VR system workflow

**Figure 5:**
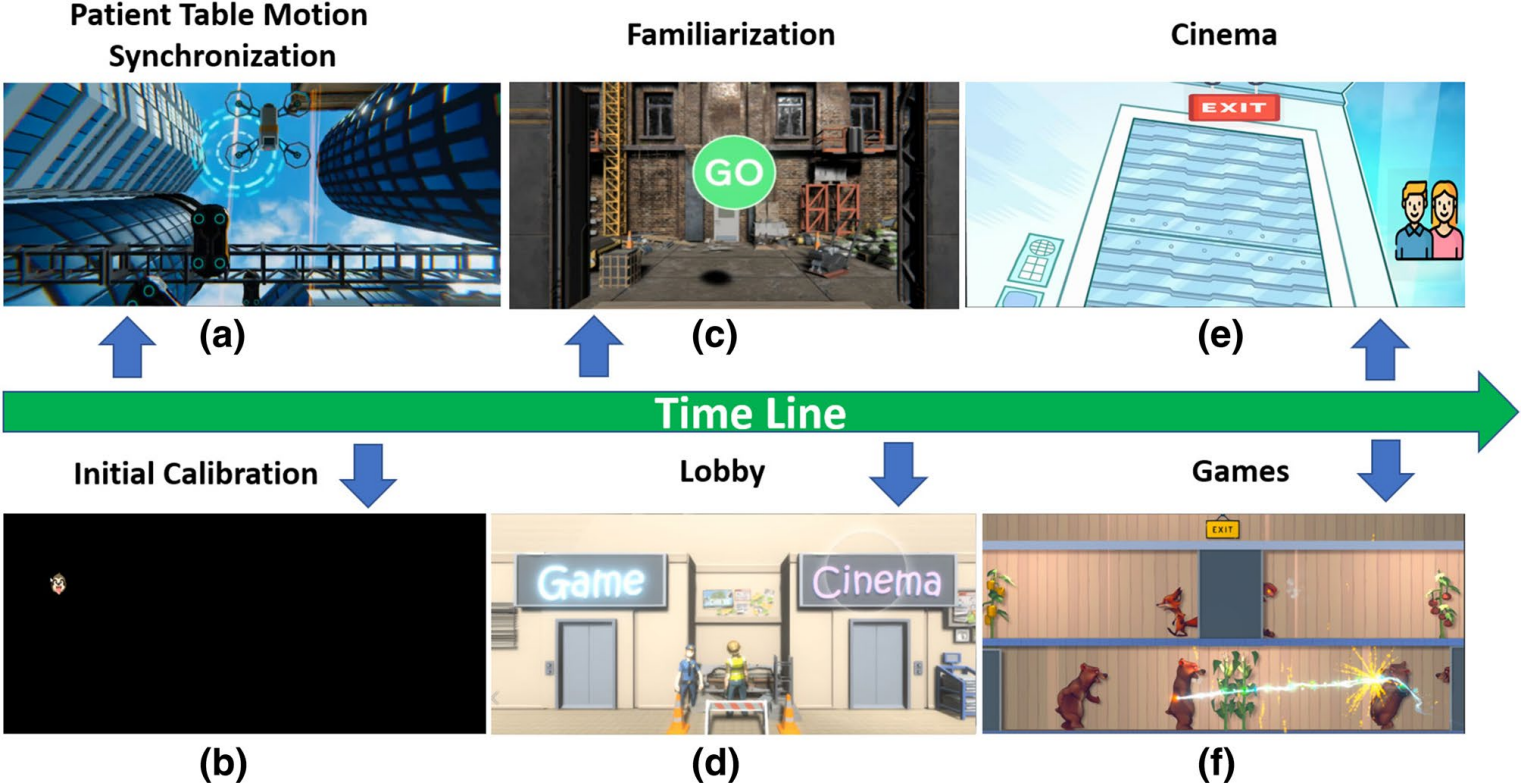
System timeline and content overview: (**a**) opening scene with frame of reference that moves to match what the subject perceives as the patient table is moved into the scanner. (**b**) Once the subject is inside the bore, the eye tracking calibration begins. (**c**) A familiarization scene follows to enhance the gaze tracking by progressive calibration. (**d**) The subject is guided into a VR lobby to select movies (**e**) and games (**f**) using gaze-based selection.

As soon as the subject is positioned on the patient table with their head inside the MRI receiver coil and the VR display system is in place, they are presented with a visual scene that is congruent with lying supine looking upwards (Fig. 5a). The operator checks the images sent by the eye cameras, manually places the required eye corner marks and verifies that robust pupil extraction is occurring. During final examination preparation, as the patient table is moved to the required location inside the scanner bore, the presented scene elements move so that the visual scene matches sense perception (care is taken to prevent the subject from touching the magnet bore as the table moves). Once the patient table stops, the 3D scene fades to black and calibration begins (Fig. 5b, see prior text). After this period with no visual cues related to orientation, which lasts 27 s, the visual perspective changes to an upright pose so the patient can navigate autonomously through a virtual space (Fig. 5c). A industrial street scene is used to establish the new spatial perspective and provide gaze control training for view control/navigation, UI interaction and game interaction. As previously described, to create congruence with scanner acquisition noise, construction site and workers (with gaze activated interaction) have been integrated into various scenes (Fig. 5c,d). The VR system initially creates their work noise, which is then blended into native scanner sounds. After exploring and interacting with the initial scene that subject passes through a door and transitions to the lobby scene (Fig. 5d). From then on, the subject is in control through gaze-based interactions supported by ongoing progressive calibration to maintain performance. Currently, the system has two categories of content: movies and games (Fig. 5e,f). The transition of each category is linked by a sliding camera animation to create an immersive 3D experience. While at the cinema or between levels during games the subject can fixate on a parent/carer call icon (Fig. 5e) to initiate an interactive session. The carer has their own display which shows whatever the subject is currently seeing plus the eye camera live stream. The carer can use a conventional mouse interface to assist the subject in the scanner if needed.

## Progressive calibration based gaze interface tailored game design

In modern games, gaze interaction is mainly used as an auxiliary input modality to provide an enhanced and more immersive user experience. A detailed introduction of eye tracking control in games can be found in^54^. From a visual perspective, gaze can be used in this context for a variety of functions including: enabling extended view, foveated rendering, dynamic UI (dynamic transparency, brightness, display etc.), dynamic light adaptation and changing the depth of field. In UI interaction, gaze can be used for operations such as menu navigation, auto pause, and zooming in. For game mechanics, it can also be used for players to aim, select, throw, fire and navigate. However, the majority of these features have evolved to work with existing games which were not initially designed to rely on eye tracking. In contrast, in our scenario, gaze is the only input for the user interface and it is critical that it remains stress free. Recently, a game named “Before Your Eyes”^55^ that uses blinking as the only input to drive a narrative adventure has been published. It successfully opens up a new game style that solely relies on eyes. However, it uses a mixture of deliberate and inadvertent blinking and the stress involved in either achieving or inhibiting these is part of the experience, which is contrary to the stress free capability for sustained use that we require.

The principle of game design is to make full use of our gaze interaction interface to achieve in-game element interaction and game control. To distinguish between the interaction and activation of specific commands, we categorize the interactions in game into immediate interactions and dwell-based interactions. The immediate interactions can be achieved by setting the dwell threshold to zero, and this is used to trigger responsive behaviour in games (such as character interaction, highlighting effect, target shooting etc.). Dwell based interactions are mainly used to activate commands for game logical control (such as reward collection, restart, continue, quit etc.). These latter events all trigger progressive calibration to maintain and enhance the robustness of gaze control. To avoid the Midas Touch problem^18^ and enable smooth game experiences, a balance is made between the gathering of visual information, immediate interaction and dwell-based interaction when designing games. For example, a tower defense game has been developed with multiple rounds requiring increasing levels of performance. In each round, the subject needs to perform immediate enemy tagging, dwell-based reward collection, trap placement and in game decision making. The interface not only retains the fun of the game but also enhances the performance of gaze estimation.

## Experiments

**Figure 6:**
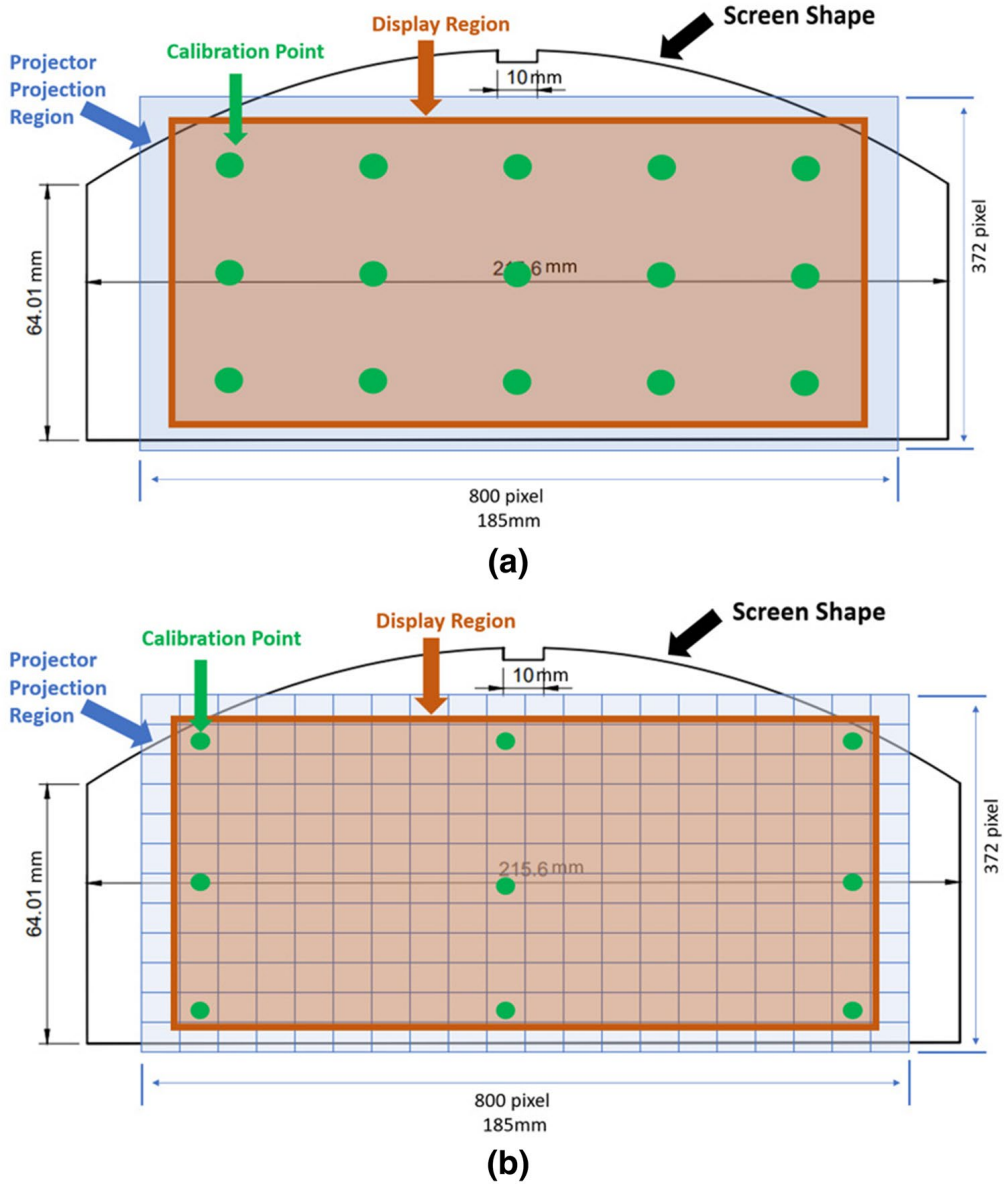
Illustration of the viewing screen shape, projection region and calibration point patterns for experiments. The green circles are targets used for calibration. (**a**) Calibration pattern for regression model comparison. (**b**) Pattern used for initial system calibration and locations of targets used for accuracy analysis (one target shown at the centre of each rectangle within display region).

The following tests were performed to specify and evaluate the performance of the gaze estimation engine. To determine the optimal regression model to deploy, a single subject fixated on a sequence of 15 static targets repeated for 8 rounds. The fifteen targets were distributed on the screen in three rows of five equally spaced locations (Fig. 6a). 15 was chosen as the most complex regression model we compared had 15 coefficients to determine. After each round of 15 points was complete, the subject changed head pose. Initial model calibration was done using recordings from the first round/first pose, and the remaining 7 poses were used for model performance testing. The model that provided the best gaze estimation was then adopted and its performance tested by 13 subjects (ages 25–45 years, 5 female, 8 male) who were asked to first fixate on 9 calibration points as shown in Fig. 6b, then a further 180 static targets were presented sequentially at 1 s intervals using rows 2–11 and columns 2–19 in the same figure. The subjects were asked to relax in one comfortable pose and focus on the presented targets only. Finally, a provocation test was performed in which one subject deliberately moved their head as much as they could during a prolonged examination period of 19 min while triggering the UI on 379 targets. In all these experiments, calibration targets remained on the screen for 3 s each.

In all these tests the data was collected and processed as follows: based on the timing of target presentation, values for $\mathbf{g} ^t$ and $\mathbf{e} ^t$ were collected and used to populate equation (1) using the current model under test. The data was processed stepwise, with a new row added to $\mathbf{G}$ and $\mathbf{V}$ at each step. At a given step, *t*, the gaze location was estimated using all of the available data to produce a regression gaze estimate, and also estimated by omitting the current data value of $\mathbf{g} ^t$ (i.e. using a model based on progressive calibration up to $t-1$) to generate a prediction gaze estimate. Gaze location was also estimated using only the initial calibration data, providing a fixed model gaze estimate. The eye corner tracking data was used to monitor head position for each gaze fixation event. These data were then used in conjunction with the actual values of $\mathbf{g} ^t$ to calculate a regression error, prediction error, fixed model error and relative motion since the previous measurement. All were specified as absolute (magnitude) distances, expressed as percentages of the screen width, providing a dimensionless measure that directly relates to the accuracy within the available field of view with which a target can be localized.

To get initial feedback on the use of gaze to control the system and understanding of the purpose of the design, 22 volunteers (ages 20–55 years) were asked to complete a full pass through the available VR content. This involved navigation through the virtual realm, exploration of all modules, including playing a game, selecting a film in the cinema and exiting each task. The system was also used in part or full by 6 children (aged 7–11). Verbal feedback was collected from all volunteers.

**Figure 7:**
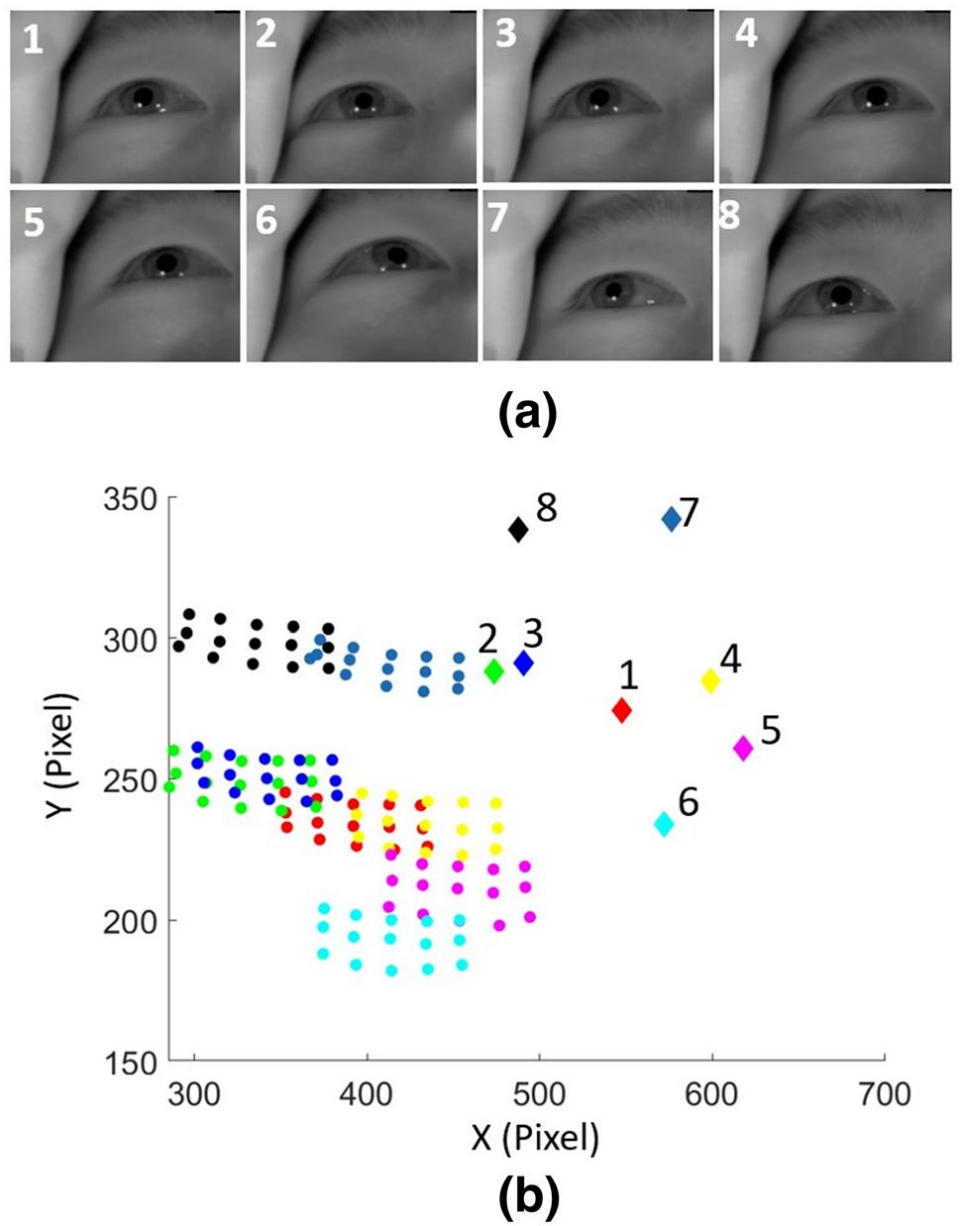
Effect of head movement on detected pupil locations when fixating on an unchanging grid of 15 points shown in Fig. 6a: (**a**) Screenshots showing eye positions in each of 8 head poses. (**b**) The pupil positions (solid circles) and eye corner locations (solid diamond) for each of the poses in (**a**). Each color group (15 pupil points and 1 median corner point) are the measured pixel values for correspondingly numbered pose. The input eye image is 640 $\times$ 480 pixels. Calibration is based on the first pose.

Ethical approval for the work was attained by the King’s College London Research Ethics Committee (HR17/18-6792) and all studies were performed in accordance with the KCL guidelines/regulations and the Declaration of Helsinki. All methods were carried out in accordance with relevant guidelines and regulations. All experimental protocols were approved by King’s College London. Informed consent was obtained from all participants, and from a parent and/or legal guardian if the participant was under 18 years of age. The participants recognizable in figures (Dr Kun Qian) and in the supplementary video (Dr Kun Qian and Dr Tomoki Arichi) are from the authorship team and have consented for the inclusion of their images in an open access publication.

## Results

**Figure 8:**
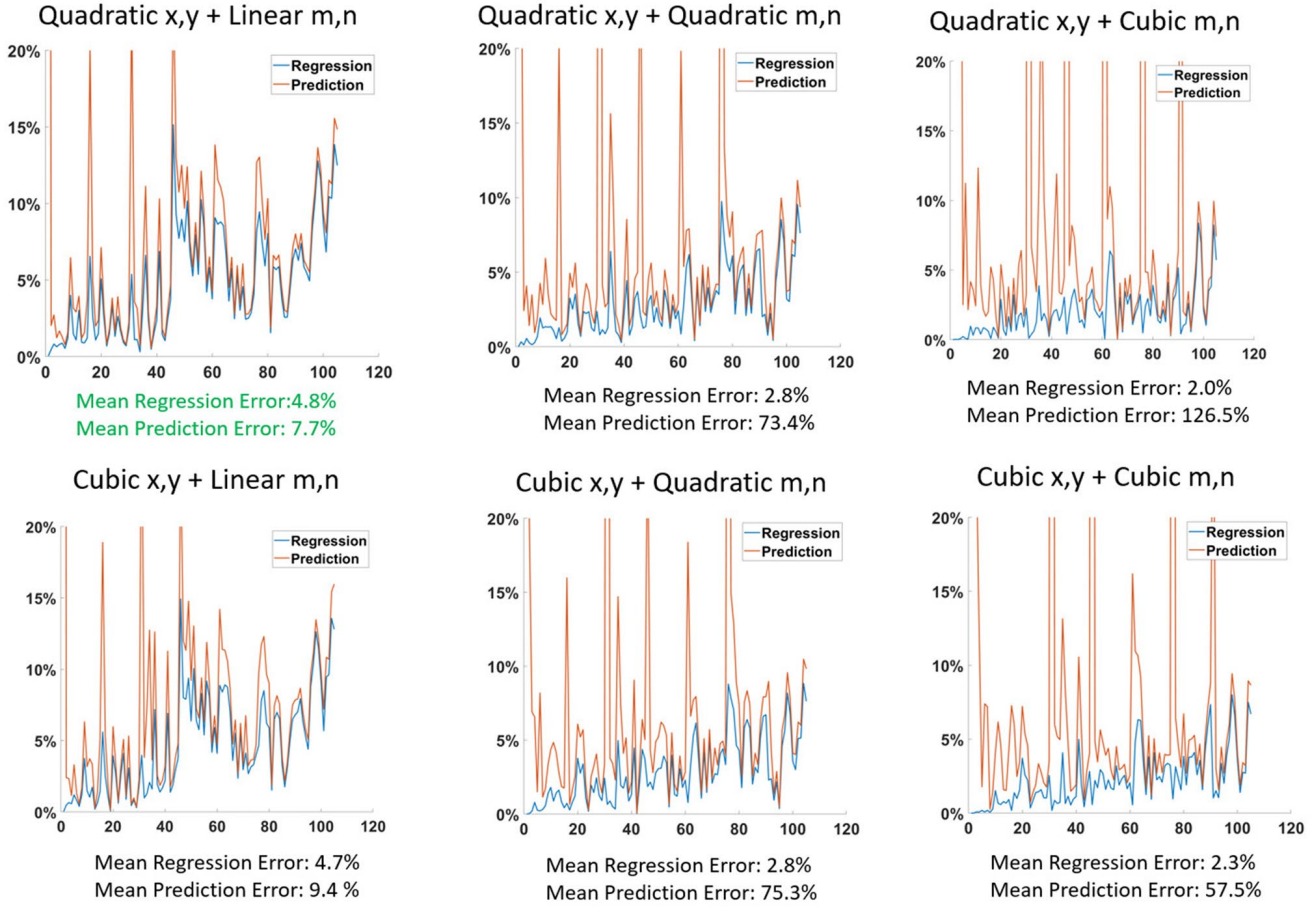
Prediction and regression errors for different models based on measurements from 7 different poses. In each graph the horizontal axis represents the sequentially numbered target fixations and the vertical axis shows the fractional regression and prediction errors as percentages of the full screen width. The mean over the full trial for regression and prediction errors for each model are shown under each graph. The smallest mean regression error is obtained with largest model, but the smallest prediction error is obtained for the model that is quadratic in pupil coordinates and linear in eye corner coordinates.

Figure 7 shows captured eye images together with measured pupil and eye corner coordinates for the model selection experiment. The patterns of coordinates change in both absolute and relative positions between poses, indicating the challenge presented for achieving a fixed model. Figure 8 shows the progressive prediction and regression errors for the models that were tested. For each model the mean regression and prediction error is also presented. As would be expected the higher order models produce the smallest regression error (minimum error 2.0%), but the quadratic model with linear terms for the eye corner positions produced the lowest prediction error (7.7%, compared to 126.5% for the model that was best for regression). Thus this model was adopted.

**Figure 9:**
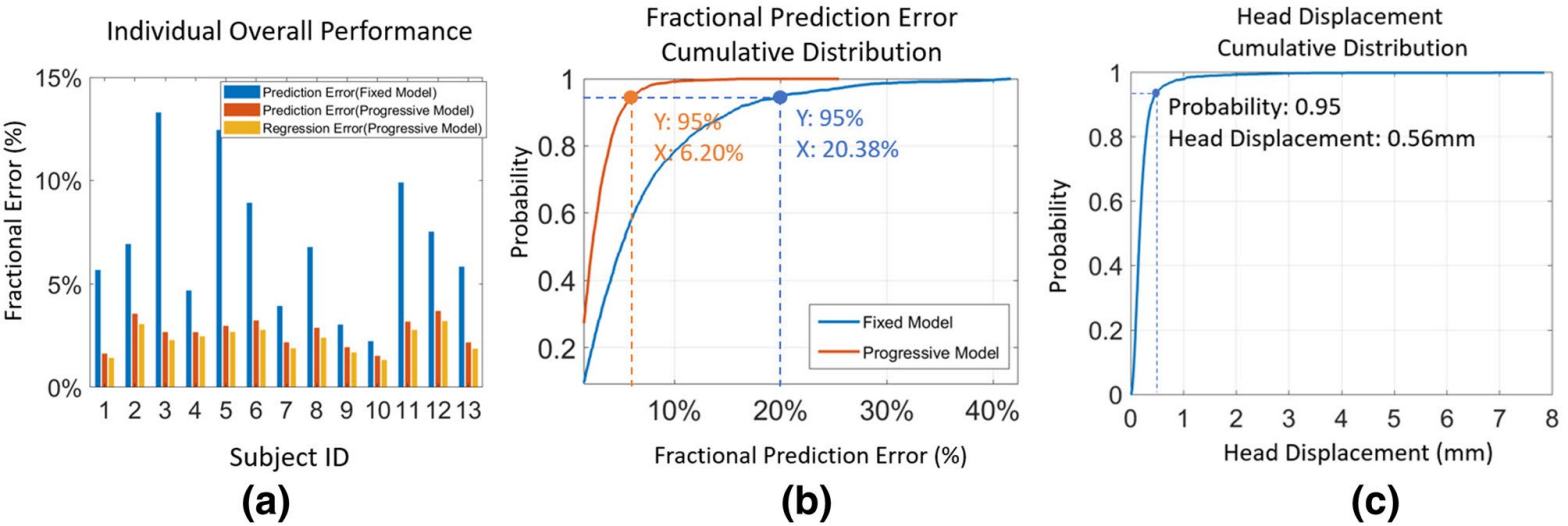
Gaze system performance: (**a**) the individual performance graph showing the mean absolute error (MAE) of prediction and regression of all targets for each subject. (**b**) The cumulative distribution for prediction errors for all targets for all subjects (progressive calibration in orange, fixed model in blue). (**c**) Cumulative distribution of the head displacement since previous fixation for all subjects.

The system accuracy test results for all 13 subjects are shown in Fig. 9. In all cases the fixed gaze model produced much larger errors than when using progressive calibration (Fig. 9a). As shown in Fig. 9b the adaptive gaze prediction error was less than 6.20% of the screen width for 95% of all estimations. This compared to over 20% for the fixed model, despite the fact that for 95% of gaze estimations the subject moved by less than 0.56 mm since the previous gaze fixation (Fig. 9c). This confirms that the progressive system is robust and reliable.

**Figure 10:**
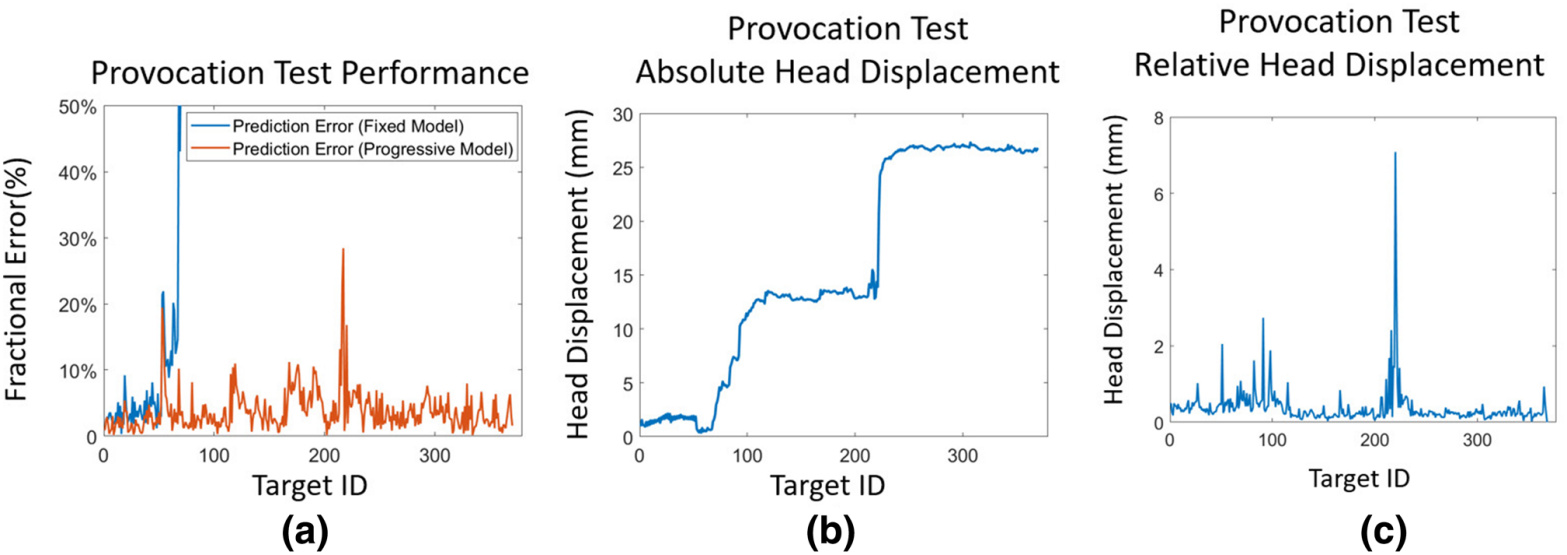
Provocation test results: (**a**) prediction errors for the fixed model (blue) and progressive model (orange). (**b**) Absolute head displacement from the pose at the first fixation event. (**c**) Relative head displacement since previous fixation. Note that a really large sudden movement (large vertical spike in (**c**) produces a corresponding larger prediction error in (**a**), but the system accuracy rapidly recovers again.

Results from the provocation test are shown in Fig. 10. Because of the extreme motion (Fig. 10b), the static model failed completely early in the trial. Although there could be up to 30% errors for the adaptive model immediately following a sudden extreme movement (Fig. 10a,c), the system then recovered and returned to the expected typical performance. By analyzing the cumulative distribution for prediction error and the relative head displacement, we found 95% of the gaze predictions have less than 8.23% error (compared to 6.20% in group test) and 95% of relative head motions are smaller than 0.93 mm (compare to 0.56 mm in group test). Although the 95 percentile cumulative prediction error increased in the provocation test compared to group test, the small increase shows that the system still remains reliable.

According to the above analysis (95% of fixations within a radius of 6.20% screen width). Using this result to define a minimum distance between targets leads to the conclusion that robust selection can be achieved for an array of 7x3 distinct targets within the visual scene, suggesting that dense gaze control capabilities are feasible for the system.

All subjects were able to finish the test task that included all aspects of the VR system. The 16 subjects with normal eyesight were immediately able to operate the UI reliably. The 6 subjects who normally use corrective lenses needed to practice in the lobby before they achieved reliable selection events, but all were able then to complete the whole test successfully. The latency of the eye tracking system is around 90ms (compared to 45–81 ms in HTC Vive Pro Eye^56^). All the participants gave verbal feedback that they were impressed and even shocked by the immersive experience achieved. The bed motion tracking was particularly remarked upon, with subjects stating that the sense experience seemed totally natural and left them without any impression of having entered the bore of the scanner despite knowing that this had happened through prior familiarity with the MRI examination process. Game playing was described as more diverting than film watching, but given the subjects were aware they were testing a new system, this is perhaps not surprising. It is interesting to find that no participants yet asked for selection support from carer as they immersed themselves in exploring the system. No motion sickness, headache or other discomforts were reported. No MRI data was acquired during these tests, as the focus was on system testing and observing the volunteers. All of the children in the preliminary tests reported positive experiences of using the system, were able to grasp the concept of gaze based interaction without additional training, and could operate the UI without difficulty. However due to their age range, varied background and the limited scope of testing to date, we do not draw any conclusions beyond feasibility of use with children from this preliminary feedback.

## Comparison with state-of-the-art research

To compare with state-of-the-art results, we compared setup complexity, accuracy, head motion range, pros and cons and potential for use with MR compatible VR with conventional non-invasive (closed and remote) eye tracking methods and commercial systems used in MRI (see Table 1). For the methods that do not describe specific head motion ranges, we categorized the head motion range into 3 levels: free, constrained and fixed where “free” indicates that the subject can move their head as they do in normal life; “constrained” means that subject head movement is constrained by the corneal reflection (which means that the subject needs to cooperate to keep the reflection on their corneal surface); and “fixed” requires the subject to keep their head as still as possible.

To assess accuracy, we averaged the 13 subjects’ prediction errors from the accuracy test and converted it to angle and pixel metrics for easy comparison with other methods. This demonstrated that the performance of our system is comparable to those achieved with state-of-the-art methods, particularly other 2D regression/feature based remote methods which show higher error than our system because of their fixed regression model and poor tolerance to head movement.

The listed appearance based methods include popular gaze tracking datasets in natural environments (such as MPIIGAZE^34^, EYEDiap^57^, RT-Gene^58^) and deep learning, and have low accuracies. However, these appearance based methods are highly dependent on the training dataset and at present, there is no dataset that meets our conditions and is therefore capable of producing accurate results. Although 3D model and cross ratio based methods can achieve good accuracy, they require stable ambient lighting, a complex lighting setup, and subject cooperation for stable corneal reflection. For commercial eye tracking systems for use inside MRI scanners, subjects are required to keep relatively still to maintain a reflection on the corneal surface for gaze estimation. Our method allows a larger head motion range, as we use eye corner detection rather than the corneal reflection. Although slightly less accurate in comparison to methods using corneal reflection, this allows our system to be more robust to varied lighting conditions and subject head pose whilst still achieving competitive accuracy.

Lastly, it is important to emphasize that these methods were designed for conventional eye tracking tasks rather than VR integration, as existing VR and eye tracking combinations are still limited to head-mounted devices. Our requirements are distinct as they involve a combination of a remote eye tracking system with limited field of view cameras and VR in a highly constrained environment, thus making existing solutions not suitable to use. Our solution achieves an innovative way of integrating gaze estimation based on remote eye tracking with VR using progressive updating during interaction, which not only provides natural and robust interaction but also high accuracy.

## Conclusion and future work

In this paper, we have proposed a non-intrusive eye tracking based MR compatible virtual reality system with bespoke hardware, custom designed user interface and content. This system demonstrates a capability to bring an interactive VR world into MRI systems. The completely non-intrusive and contactless design does not require special preparation work before the scan (such as sticking markers to the subject’s face). The progressive calibration-based eye tracking mechanism provides a robust control interface for the VR system, with testing confirming that the system can achieve gaze-based selection and interaction for basic VR requirements. Although currently VR content is limited, all subjects including both adults and children gave positive feedback about their experiences. The characteristic of our progressive calibration gaze interface can be easily extended to many existing games as the core control and interaction mechanisms are the same. Meanwhile, gaze based control can open up a new game style for motion restricted subjects (such as patients with motor disorders etc). A notable finding was that the system was highly effective at removing the sense of being inside the MRI scanner bore, which could be a substantial benefit to those who suffer from anxiety and claustrophobia. This was achieved by completely replacing the visual scene and by seeking to create a congruence between the virtual world and the other sensations that are perceived during MRI examinations. So far this has been done by including elements in the visual scene that indicate building works are in progress in the virtual world, which could account for scanner noise and vibration. Construction sounds are blended with scanner noise to maximise congruence. There is potential for a greater level of integration, but to achieve this would require close coupling of VR content with scanner operation. Since MRI examinations can vary widely and it is not uncommon to adaptively adjust protocols during examinations, such close integration was deemed to be beyond the scope of initial system development. Clearly much more in-depth testing of the current system is needed, including proper integration with full MRI examinations and systematic studies with children.

The proposed system has both clinical applications for subjects who find MRI stressful (such as those with claustrophobia, subjects with impaired cognitive function and children) and for neuroscience with (as yet untested) potential as a platform for a new generation of “natural” fMRI experiments studying fundamental (but hitherto poorly understood) cognitive processes such as social communication. The adaptive gaze tracking and control system proved robust and subjects found it intuitive and comfortable to use. This custom designed VR system for enhancing MRI acceptability has received positive feedback from all participants who have tried it, which encourages development towards clinical practice and as a research platform.

**Table 1:** Comparison with state of the art methods.

Category	Method	Setup	Accuracy (in degree or pixels)	Head motion range	Limitation	Advantage	Potential for MR compatible VR
2DRFB	Ours	2 Cameras with 1 LED each	0.76$^{\circ }$ /21.16pixels	30 mm	1. Gaze interaction is required to maintain accuracy when large head motion happens. 2. Close eye image required	1. Accurate for relative fixed head motion. 2. No need for corneal reflection (robust to head motion)	High
2DRFB	Su et al.^59^ (2020)	1 Camera	77.46 pixels	Unconstrained	1. Poor accuracy for large head motion	1. Fair accuracy for fixed head	Low
2DRFB	Arar et al.^25^ (2016), Shin et al.^23^ (2015), Ma et al.^26^ (2015)	1–2 Cameras + light array.	0.91$^{\circ }$–2.50$^{\circ }$	Constrained	1. Corneal reflection required 2.Vulnerable to head motion	1.Accurate for fixed head	Low
2DRFB	Chi et al.^24^ (2009)	1 Camera + light array	20.00 pixels	Constrained	1. Corneal reflection required 2.Vulnerable to head motion	1. Accurate for fixed head	Low
AB	Wood et al.^33^ (2016), Zhang et al.^34^ (2017), Cheng et al.^35^ (2020)	1 Camera	4.41$^{\circ }$–9.95$^{\circ }$	Free	1. Poor accuracy. 2. Rely on dataset.	1. Calibration free. 2. Low hardware requirement	Low
AB	Kim et al.^60^ (2019)	Near eye camera + infrared light	3.51$^{\circ }$	Fixed	1.Poor accuracy. 2. Rely on dataset.	1. Calibration free. 2. Low hardware requirement	Low
3DMB	Sigut et al.^27^ (2010) Hennessey et al.^28^ (2006) Beymer et al.^29^ (2003)	1–4 Cameras + light array	0.60$^{\circ }$–3.00$^{\circ }$	Constrained	1. Complex setup. 2. Corneal reflection required.	1. Accurate and head motion allowed	Low
CRB	Coutinho et al.^30^ (2006) Huang et al.^31^ (2014) Zhang et al.^32^ (2014)	1 Camera + light array	0.60$^{\circ }$–2.00$^{\circ }$	Constrained	1. Vulernable to user distance variance. 2. Corneal reflection required. 3. Complex setup	1. Accurate and head motion allowed	Low
MRCETS	MRC Eye Tracking Solution (MRC)^61^	Coil mount + 2 LEDs	0.40$^{\circ }$	Not mentioned	1. Corneal reflection required. 2. Close eye image required	1. Accurate for relative fixed head movement	Low
MRCETS	LiveTrack AV (Cambridge Research System)^62^	Coil mount + 2 infrared lights	0.50$^{\circ }$	20 mm	1.Corneal reflection required. 2. Close eye image required	1. Accurate for relative fixed head movement	Low
MRCETS	VisualSystem HD (NordicNeuroLab)^63^	Near face coil mount + 2 infrared lights	0.20$^{\circ }$–0.50$^{\circ }$	Fixed	1. Corneal reflection required. 2. Close eye image required	1. Accurate for relative fixed head movement	Low
MRCETS	DeepVOG^21^ (2019)	Near face coil mount + 2 infrared lights	0.50$^{\circ }$	Fixed	1. Corneal reflection required. 2. Close eye image required	1. Accurate for relative fixed head movement	Low

We categorize these methods into: 2DRFB = 2D Regression/Feature Based; AB = Appearance Based; 3D Model Based = 3DMB; Cross Ratio Based = CRB; MR Compatible Eye Tracking Software = MRCETS.

## Supplementary Information

Supplementary Video.

Supplementary Information.
