# Supplementary material for: An eye tracking based virtual reality system for use inside magnetic resonance imaging systems
Source: Sci Rep. 2021 Aug 11;11:16301. doi: 10.1038/s41598-021-95634-y (PMC8357830; doi:10.1038/s41598-021-95634-y)
Supplement: Supplementary file 2 — Supplementary Information. [file 41598_2021_95634_MOESM2_ESM.docx]

Supplementary Video S1 legend: the supplementary video demonstrates how the system works, with the following elements: patient table motion synchronization from 0:00s to 0:28s; calibration for eye tracking from 0:29s to 0:41s; familiarization content from 0:42s to 4:06s; lobby/game/cinema experience from 4:07s to 6:22s; patient removal from 6:23s – 6:41s. Please note the soundtrack has been removed from the video for copyright reasons.
